# Hemorrhagic varices around the pancreatojejunostomy site due to left‐sided portal hypertension after pancreaticoduodenectomy: A case report

**DOI:** 10.1002/deo2.397

**Published:** 2024-06-17

**Authors:** Yasuaki Abe, Tatsuhide Nabeshima, Kazuhiro Sakuta, Yuko Nishise, Michio Kuroki

**Affiliations:** ^1^ Department of Gastroenterology Yamagata City Hospital Saiseikan Yamagata Japan

**Keywords:** cyanoacrylates, endoscopic hemostasis, hemorrhagic varices, pancreaticoduodenectomy, sinistral portal hypertension

## Abstract

Pancreaticoduodenectomy (PD) with combined portal vein resection sometimes causes left‐sided portal hypertension, which can be a problem. An appropriate treatment strategy for hemorrhagic ectopic varices due to left‐sided portal hypertension after PD has not yet been determined. We report a case of repeated variceal rupture around the pancreatojejunostomy site. A 65‐year‐old woman with a history of PD for pancreatic head cancer was admitted with a chief complaint of bloody stools. She was diagnosed with pancreatojejunostomy variceal rupture, and an endoscopic cyanoacrylate injection was performed. As rebleeding occurred 2 weeks after the first treatment, endoscopic cyanoacrylate injection was repeated, and hemostasis was achieved. Additionally, she had esophageal, colonic, and gastrojejunostomy varices, and the future risk of these variceal ruptures was considered very high. Hence, a splenectomy was performed to prevent rebleeding or other variceal ruptures. Endoscopic cyanoacrylate injection is a useful treatment for hemorrhagic varices around the pancreatojejunostomy site. It is also necessary to understand portal vein hemodynamics and provide appropriate additional treatment in cases of recurrent variceal rupture due to left‐sided portal hypertension after PD.

## INTRODUCTION

Pancreatic head cancer sometimes infiltrates the portal vein (PV), and combined PV resection with pancreaticoduodenectomy (PD) is required to achieve negative‐margin resection. The splenic vein (SpV) is sometimes ligated during PD and ectopic varices including pancreatojejunostomy varices occur due to left‐sided portal hypertension (LSPH) after PD.

There are only a few reports of these cases and only a few reports of bleeding cases. Therefore, a treatment strategy for hemorrhagic ectopic varices after PD has not yet been established. The usefulness of endoscopic cyanoacrylate injection is unclear, and the need for additional treatments to improve LSPH, such as splenectomy or partial splenic embolization, is unknown.

In this report, we describe a case of hemorrhagic varices around pancreatojejunal anastomosis due to LSPH after PD that was treated by endoscopic cyanoacrylate injection and splenectomy.

## CASE REPORT

A 65‐year‐old woman presented with hematochezia. She had undergone PD with PV resection for pancreatic head cancer 45 months before. The cancer had infiltrated the PV‐superior mesenteric vein (SMV) confluence. Neoadjuvant chemotherapy with gemcitabine and nab‐paclitaxel had been conducted. In PD, the SpV was ligated and not reconstructed; the inferior mesenteric vein, middle colic vein, and gastrocolic trunk were also divided. No complications were identified after PD. She also underwent endoscopic injection sclerotherapy with 5% ethanolamine oleate for hemorrhagic esophageal varices 43 months after PD.

**FIGURE 1:**
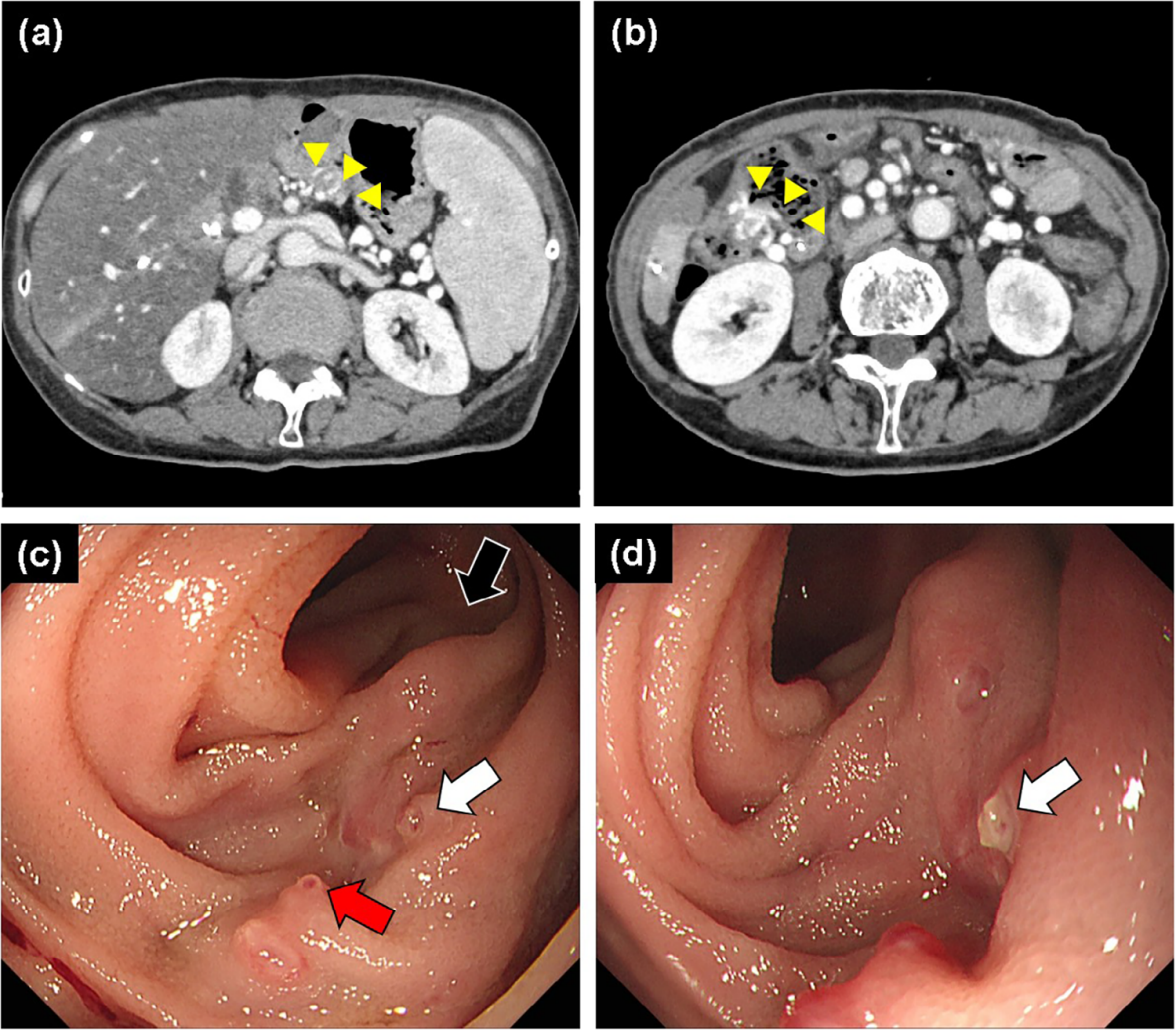
(a, b) Computed tomography: (a) pancreatojejunostomy varices (yellow arrowhead), (b) colonic varices (yellow arrowhead), (c, d) single‐balloon endoscopy reveals F2 varices with red color signs (red arrow) and white plugs (white arrow) around the pancreatojejunal anastomosis (black arrow).

Her vital signs were stable; however, laboratory tests revealed severe anemia (hemoglobin 5.9 g/dL). Contrast‐enhanced computed tomography revealed colonic, gastrojejunostomy, and pancreatojejunostomy varices (Figure 1a,b), however, the bleeding point could not be detected. No constriction was observed during the reconstructed anastomosis of the PV‐SMV. Although the colonoscopy revealed a large amount of fresh blood and varices in the ascending colon, there were no signs of rupture. Upper gastrointestinal endoscopy could not identify the source of the bleeding.

Transoral single‐balloon endoscopy (SBE, SIF‐H290S; Olympus) was performed to observe the pancreatojejunal anastomosis. SBE revealed F2 varices with a red color sign and a sign of recent bleeding (white plug) around the pancreatojejunostomy site (Figure 1c,d). Thus, 1.0 mL of 75% *N*‐butyl‐2‐cyanoacrylate (nb‐CA, Hystoacryl; B.Braun) was injected into the varices using a 23‐G, 2200‐mm long endoscopic puncture needle (Boston Scientific Corporation). Retention of the adhesive agents was confirmed using an X‐ray system. Furthermore, subsequent computed tomography showed good stagnation of the adhesive agent in the varix and no leakage into the intrahepatic PV. However, 2 weeks after the first endoscopic cyanoacrylate injection, the varices ruptured again. An SBE was performed again and revealed fresh blood in the afferent loop.

**FIGURE 2:**
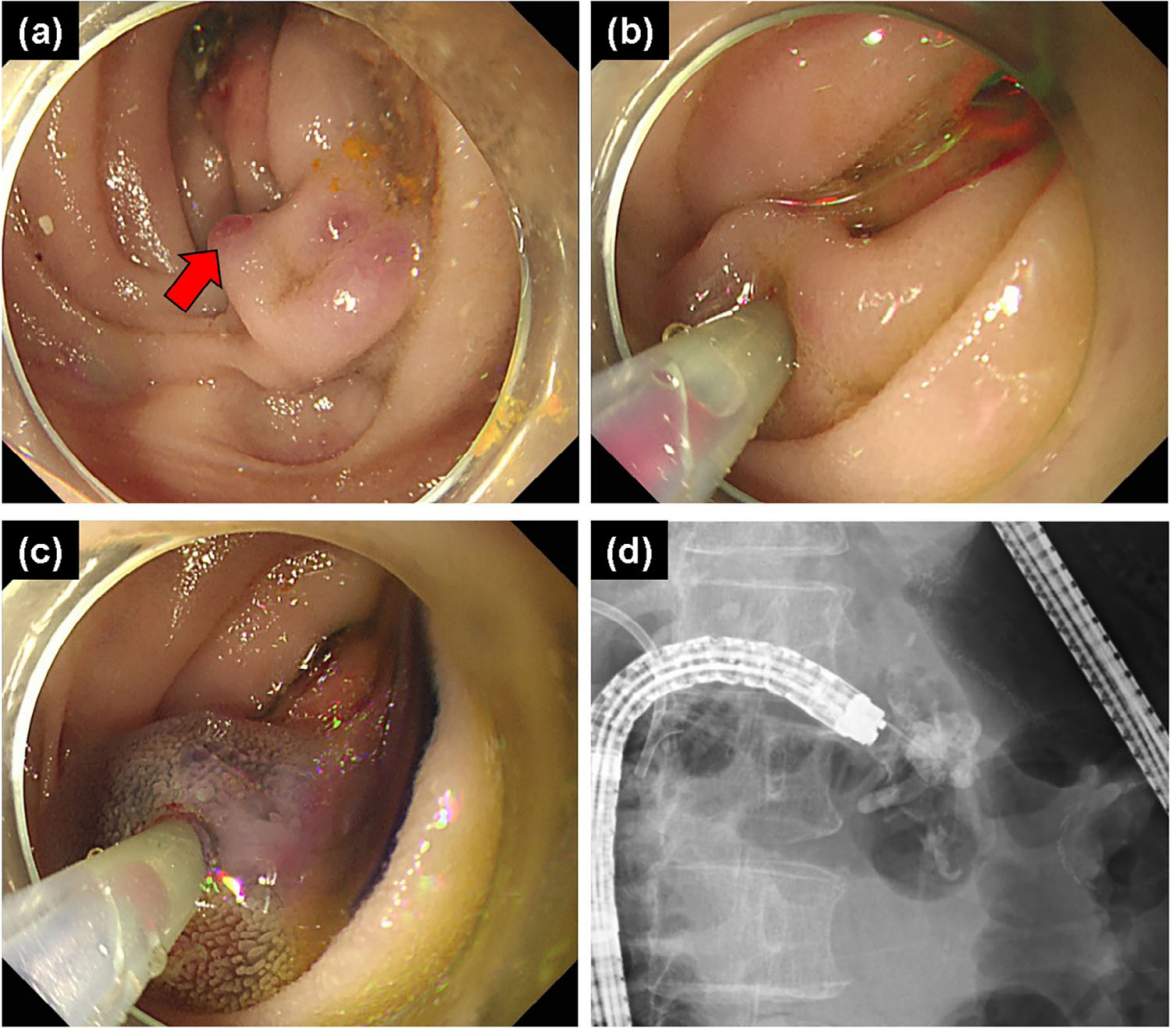
(a–c) Endoscopic images at the repeated variceal rupture, (a) varix on the oral side of the previous rupture point remains and the red color sign (red arrow) has worsened, (b, c) Endoscopic cyanoacrylate injection for pancreatojejunostomy varices is repeated, (d) the varices are filled with the adhesive agent.

Although the previous treatment was considered to be effective, the varix on the oral side of the previous rupture point remained, and the red color sign worsened. We injected an additional 1.5 mL of 75% nb‐CA (Figure 2). After the second endoscopic cyanoacrylate injection, the patient had no recurrent bleeding. The procedure time (time from the insertion of the endoscopy to its removal) for both the first and second treatments was 25 min.

In this patient, because the general condition and expected prognosis were good, additional treatment was considered to prevent rebleeding or future rupture of other varices, such as the colonic and gastrojejunostomy varices. Thus, 3 weeks after the second endoscopic cyanoacrylate injection, a splenectomy was performed to improve the LSPH.

## DISCUSSION

We made the following two important suggestions for this case. Endoscopic cyanoacrylate injection is a useful treatment for hemorrhagic varices around the pancreatojejunostomy site. It is necessary to understand PV hemodynamics and administer appropriate additional treatment after endoscopic cyanoacrylate injection in patients with repeated variceal ruptures due to LSPH after PD.

First, endoscopic cyanoacrylate injection is a useful treatment for hemorrhagic varices around the pancreatojejunostomy site. Varices around the pancreatojejunostomy site after PD are rare, and cases of bleeding are even rarer. Occasionally, the management of bleeding can be difficult, and patients may end up in a serious situation. We searched PubMed using the keywords “pancreaticoduodenectomy” and “varices” and found seven reported cases of hemorrhagic pancreatojejunostomy varices due to LSPH after PD (Table 1).^1^, ^2^, ^3^, ^4^, ^5^ Although there is no standard treatment, balloon endoscopy enables the approach and treatment of pancreatojejunostomy varices. When performing endoscopic cyanoacrylate injection, it is difficult to judge how much to inject and where to puncture the varix, and care must be taken to avoid the flow of the adhesive agent into the intrahepatic PV. In this case, based on computed tomography and endoscopic images, the diameter of the varix was relatively small; therefore, we decided to inject 1.0 mL of nb‐CA near the rupture point and add more if it was insufficient, during the first endoscopic cyanoacrylate injection session. Rebleeding occurred but was controlled by the second endoscopic cyanoacrylate injection. The additional injection amount was determined based on the effects of the first treatment.

**TABLE 1:** Seven cases with hemorrhagic varices around the pancreatojejunostomy site after pancreaticoduodenectomy.

No.	Author	Year	Age/sex	Primary disease	SpVR/status of SpVR	Duration from surgery to bleeding	Treatment
1	Hong^1^	2021	59/M	Pancreatic cancer	No	15 M	IVR (percutaneous trans‐splenic vein embolization)
2	Kushiya^2^	2019	70/F	Pancreatic cancer	No	84 M	IVR (partial splenic embolization)
3	Kushiya^2^	2019	80/M	Pancreatic cancer	No	18 M	IVR (percutaneous trans‐hepatic obliteration)
4	Tanaka^3^	2019	68/F	Pancreatic cancer	No	23 M	Splenectomy
5	Tanaka^3^	2019	72/M	Pancreatic cancer	Yes/occluded	10 M	Endoscopic variceal ligation
6	Gyoten^4^	2017	62/F	Pancreatic cancer	No	6 M	Emergent splenectomy
7	Gonçalves^5^	2015	56/M	Pancreatic cancer	NA	24 M	Endoscopic cyanoacrylate injection

Abbreviations: IVR, interventional radiology; NA, not available; SpVR, splenic vein reconstruction.

**FIGURE 3:**
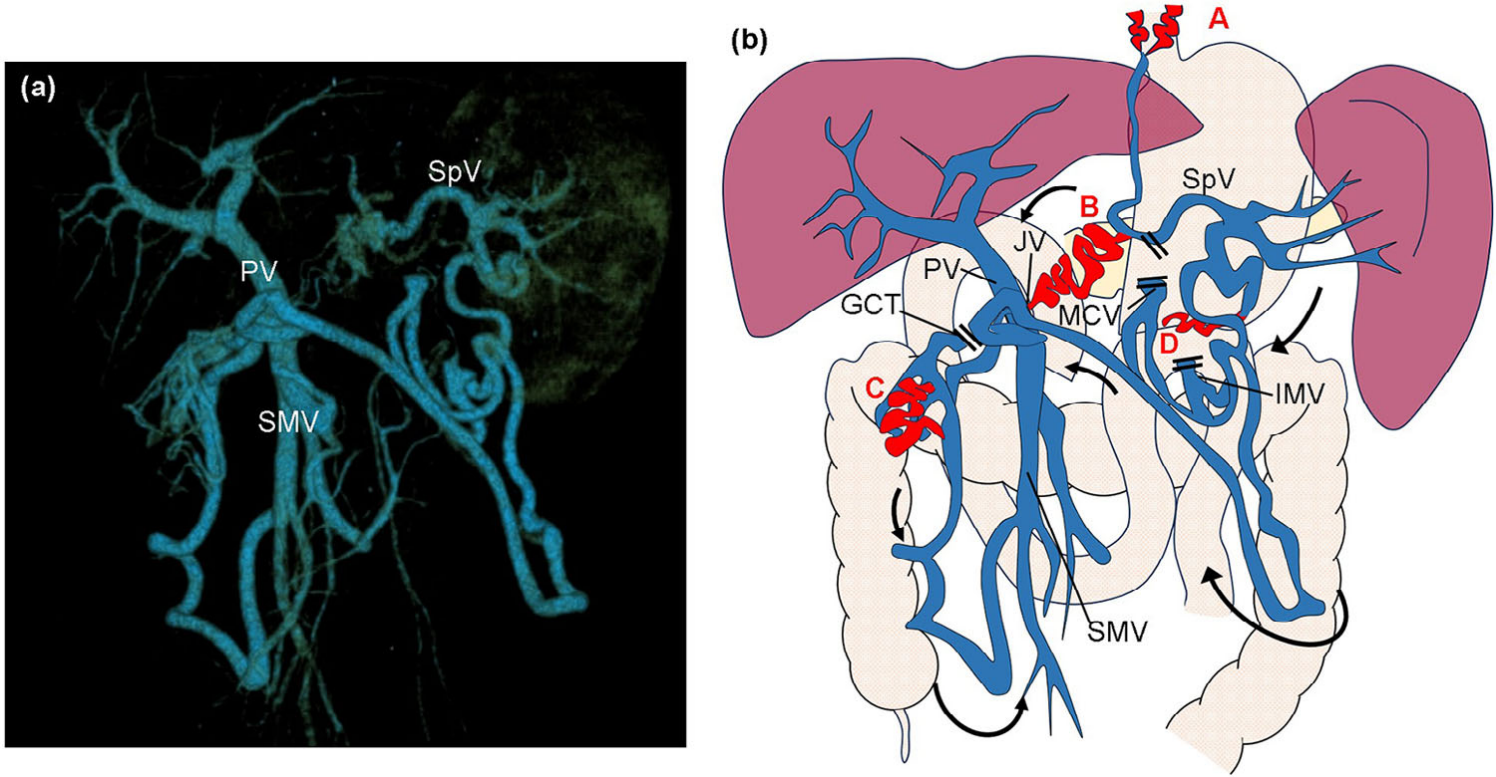
(a) Three‐dimensional vascular images, (b) schema of vascular anatomy: In the pancreaticoduodenectomy with portal vein resection, inferior mesenteric vein, middle colic vein, and gastrocolic trunk are divided with splenic vein. After the surgery, the blood from the spleen flows to the portal vein via the venous arcade of Barkow, the marginal vein along the transverse colon, the right colic marginal vein, and the superior mesenteric vein. Four varices are formed. A; esophageal varices, B; pancreatojejunostomy varices, C; colonic varices, D; gastrojejunostomy varices. Abbreviations: GCT, gastrocolic trunk; IMV, inferior mesenteric vein; JV, jejunal vein; MCV, middle colic vein; PV, portal vein; SMV, superior mesenteric vein; SpV, splenic vein.

Second, it is necessary to understand the PV hemodynamics and administer appropriate additional treatment after endoscopic cyanoacrylate injection. In patients with pancreatic head cancer that invades the PV or SMV, extended PV resection with PD is acceptable and reasonable because of improvements in margin‐negative resection and survival rates.^6^, ^7^ Although the SpV is sometimes divided during PD, whether it is reconstructed remains controversial. Furthermore, in extended PD for infiltrative pancreatic head cancer, the inferior mesenteric vein, middle colic vein, and gastrocolic trunk can also be divided. As a result, blood from the SpV flows to the PV via the venous arcade of Barkow, the marginal vein along the transverse colon, the right colic marginal vein, and the SMV. Owing to these hemodynamic changes, four varices are formed after PD with SpV ligation: pancreatojejunostomy, esophageal, colonic, and gastrojejunostomy varices^8^ (Figure 3). Because this patient had all four of these varices and not only rupture of esophageal varices but also repeated rupture of pancreatojejunostomy varices occurred, the future risk of other variceal ruptures was considered to be very high. Therefore, we investigated splenectomy and partial splenic embolization as treatments for LSPH. In a report comparing splenectomy and partial splenic embolization in improving hypersplenism, splenectomy was reported to be preferable when the splenic volume was under 400 mL.^9^ In this case, the splenic volume before PD was 190 mL and the volume after PD was 330 mL. Furthermore, because the patient's general condition was good and a good prognosis was expected, splenectomy was performed. Although it is unclear whether additional treatment to improve LSPH should be performed in all cases with variceal rupture, it should be considered in patients with a high risk of rebleeding and in good general condition. Furthermore, endoscopic cyanoacrylate injection is considered a good choice for primary hemostasis until additional treatment.

In conclusion, endoscopic cyanoacrylate injection proved useful for treating hemorrhagic varices around the pancreatojejunostomy site. Understanding PV hemodynamics and appropriate additional treatment after endoscopic cyanoacrylate injection is necessary in patients with repeated variceal ruptures due to LSPH after PD.

## CONFLICT OF INTEREST STATEMENT

None.

## ETHICAL STATEMENT

None.
